# Discovering Hair Biomarkers of Alzheimer’s Disease Using High Resolution Mass Spectrometry-Based Untargeted Metabolomics

**DOI:** 10.3390/molecules28052166

**Published:** 2023-02-25

**Authors:** Yu-Hsiang Su, Chih-Wei Chang, Jen-Yi Hsu, Shih-Wen Li, Pi-Shan Sung, Ru-Hsueh Wang, Chih-Hsing Wu, Pao-Chi Liao

**Affiliations:** 1 Division of Neurology, Department of Internal Medicine, Ditmanson Medical Foundation Chia-Yi Christian Hospital, Chiayi City 60002, Taiwan; 2 Department of Environmental and Occupational Health, College of Medicine, National Cheng Kung University, Tainan 704, Taiwan; 3 Department of Neurology, National Cheng Kung University Hospital, College of Medicine, National Cheng Kung University, Tainan 704, Taiwan; 4 Department of Family Medicine, National Cheng Kung University Hospital, College of Medicine, National Cheng Kung University, Tainan 704, Taiwan; 5 Institute of Gerontology, College of Medicine, National Cheng Kung University, Tainan 701, Taiwan; 6 Department of Food Safety/Hygiene and Risk Management, College of Medicine, National Cheng Kung University, Tainan 701, Taiwan

**Keywords:** Alzheimer’s disease, untargeted metabolomics, hair, biomarker discovery, high-resolution mass spectrometry

## Abstract

Hair may be a potential biospecimen to discover biomarkers for Alzheimer’s disease (AD) since it reflects the integral metabolic profiles of body burden over several months. Here, we described the AD biomarker discovery in the hair using a high-resolution mass spectrometry (HRMS)-based untargeted metabolomics approach. A total of 24 patients with AD and 24 age- and sex-matched cognitively healthy controls were recruited. The hair samples were collected 0.1-cm away from the scalp and further cut into 3-cm segments. Hair metabolites were extracted by ultrasonication with methanol/phosphate-buffered saline 50/50 (*v*/*v*) for 4 h. A total of 25 discriminatory chemicals in hair between the patients with AD and controls were discovered and identified. The AUC value achieved 0.85 (95% CI: 0.72~0.97) in patients with very mild AD compared to healthy controls using a composite panel of the 9 biomarker candidates, indicating high potential for the initiation or promotion phase of AD dementia in the early stage. A metabolic panel combined with the nine metabolites may be used as biomarkers for the early detection of AD. The hair metabolome can be used to reveal metabolic perturbations for biomarker discovery. Investigating perturbations of the metabolites will offer insight into the pathogenesis of AD.

## 1. Introduction

Alzheimer’s disease (AD) is a progressive neurodegenerative disorder related to cognitive impairments that generally include deficits in short-term memory and executive and visuospatial dysfunctions and is thought to account for 60–80% of dementia cases [1]. Physicians can diagnose AD through gradual onset of cognitive and behavioral symptoms, medical history, and a cognitive function test [2]. As cognitive deficits and behavioral abnormalities have been difficult to manifest in the early stage of AD, conducting early diagnosis and AD onset prediction, biomarkers are needed to obtain additional insights into the etiology of AD symptoms. In addition, AD dementia is difficult to distinguish from other dementia-causing pathologies since some AD symptoms are similar to those of other diseases. Progressively accumulating extracellular amyloid-beta (Aβ) plaques and intracellular neurofibrillary tangles formed by hyperphosphorylated tau protein are specific key proteins and considered two pathological hallmarks reflecting AD pathology [3]. Quantifying biomarker levels in cerebrospinal fluid (CSF) and measuring Aβ levels using positron-emission tomography imaging have been used to diagnose AD; however, it is very difficult to obtain CSF samples, and these processes are expensive for employment in the clinical practice [4,5,6].

Untargeted metabolomics in hair may be a novel approach for discovering novel diagnostic markers and developing new potential therapeutic targets in AD. Metabolomics is the study of the small-molecule composition within biological systems, and it has been applied to multiple fields [7]. The human metabolome is estimated to contain at least 220,000 compounds, and a large fraction of metabolites remain unidentified [8]. CSF and blood are the most conventional biological specimens used to identify AD metabolic biomarkers [3,9,10]. The procedures requiring collection of CSF samples are too invasive for routine clinical use. As urine and blood metabolomes are affected by circadian rhythms, daily activities, and sleep patterns, the concentrations of chemical compounds fluctuate daily [11,12,13]. Recently, the use of hair has been shown to be important for the long-term accumulation of both environmental substance exposure and endogenous metabolite perturbations and has been applied to disease biomarker discovery [14,15,16]. Hair segments of 1 cm reflect the metabolic profile over the past month, as they grow 1 cm per month [17,18]. Chemical compounds in blood capillaries connected to hair roots are incorporated into the hair via passive diffusion [19,20,21]. The longitudinal distribution of chemical compound concentrations along the hair shaft reflects changes in the corresponding composition during the measurement period [20].

The human hair metabolome might be used for discovering novel biomarkers of AD since it can reflect the metabolic profiles of body burden over several months. As distinguishing AD from other causes of dementia based on apparent symptoms is often difficult, the discovery of hair biomarkers may enable the diagnosis of AD during the early stage while monitoring its progression is an important strategy for managing the disease. Here, we performed an ultrahigh-performance liquid chromatography-high-resolution mass spectrometry (UHPLC-HRMS)-based untargeted metabolomic approach on hair samples from AD patients and subjects with normal cognition serving as controls. We investigated the perturbations in the hair metabolome between AD patients and cognitively healthy controls and further discovered tentatively metabolic biomarkers to distinguish AD patients from cognitively healthy controls.

## 2. Results

### 2.1. Study Design

**Figure 1:**
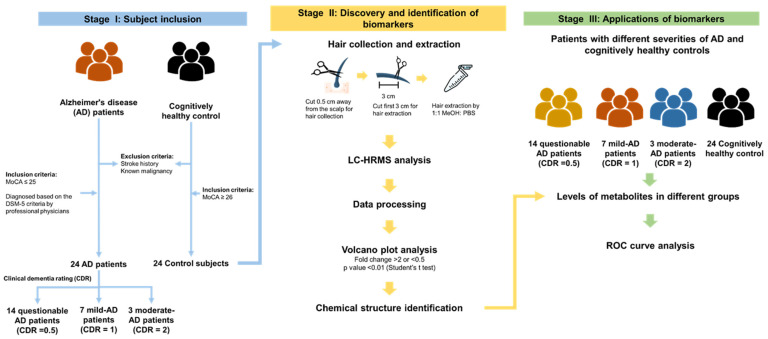
Study design of hair biomarkers of Alzheimer’s disease (AD) discovery and interpretation using a high-resolution mass spectrometry (HRMS)-based untargeted metabolomics approach. A total of 24 AD patients and 24 control subjects without stroke history and known malignancy were recruited in this study. All participants underwent MoCA test, and the control subjects included a MoCA score of higher than 26. The AD patient underwent further CDR assessment to quantify the severity of the symptoms of dementia.

The study design for discovering hair AD biomarkers is depicted in Figure 1. The participants were recruited from National Cheng Kung University Hospital, Tainan, Taiwan. Hair samples were collected 0.1-cm away from the scalp from 24 AD patients and 24 age- and sex-matched cognitively healthy controls. Hair metabolites were extracted by an established and optimized procedure described in a previous study [22]. The hair extracts were subjected to UHPLC-HRMS analysis with full scan and the raw data were processed with Progenesis QI software, which yielded a peak detection and alignment table. Fold change and Student’s *t* test were performed to identify differential levels of features. We used the available online databases of Massbank of North America (MoNA) with MS-DIAL 4.48 and MS-FINDER 3.50 software to identify the chemical structures of potential biomarkers.

### 2.2. Characteristics of the Case and Control Groups

Table 1 presents the basic demographic characteristics of 48 participants recruited from the National Cheng Kung University Hospital, Tainan, Taiwan. The case group included 24 patients with AD, and the control group consisted of 24 age- and sex-matched subjects without AD (Montreal Cognitive Assessment (MoCA) score ≥ 26). Participants with a history of stroke or known malignancy were excluded from this study. AD patients were diagnosed based on the Diagnostic and Statistical Manual of Mental Disorders, Fifth Edition (DSM-5) criteria by professional physicians from the Department of Neurology. The mean MoCA scores for AD patients and control subjects were 15.5 (SD = 5.3) and 27.4 (SD = 1.4), respectively. The severity of dementia symptoms was assessed by neurologists using the Clinical Dementia Rating (CDR) scale; fourteen patients were assessed with questionable AD (CDR score of 0.5), seven patients with mild AD (CDR score of 1), and three with moderate AD (CDR score of 2) [23]. A comparison between the demographic variables of the AD patients and controls showed no statistically significant difference in age (mean = 68.7 and 66.2 years, respectively, *p* = 0.17), body mass index (BMI) (mean = 24.2 and 23.9 kg/m^2^, respectively, *p* = 0.83), cosmetic hair treatment (*p* = 0.93), smoking status (*p* = 0.33), or alcohol consumption (*p* = 0.60).

### 2.3. Discovery and Identification of AD Biomarkers

All 3-cm hair segments were subjected to the sample preparation procedure and subsequently analyzed by UHPLC-HRMS in positive and negative ion modes, resulting in 12,358 and 8821 aligned peak features, respectively, extracted from the corresponding data. Figure S1A shows the relative standard deviations (RSDs) of the peak areas of all detected signals within the quality control (QC) sample, which was prepared by pooling aliquots from all the individual samples, resulting in a calculated median RSD of 46%; 60% of the total detected peaks had RSD values below 56%.

**Figure 2:**
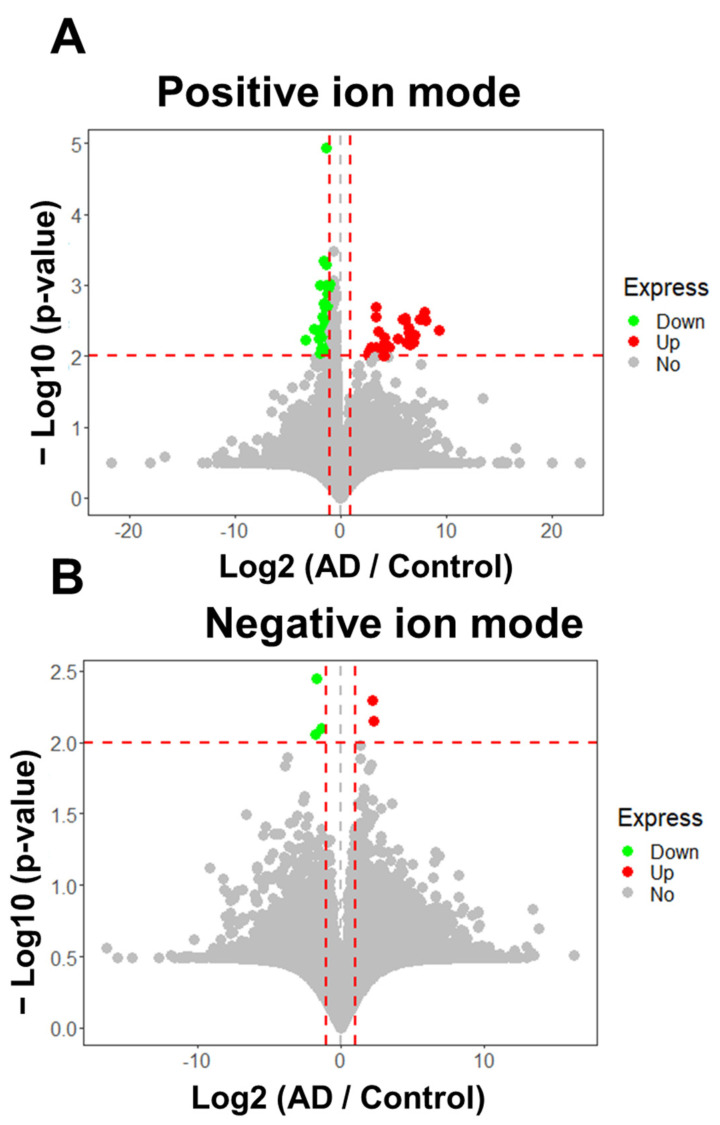
Statistically significant features (AD/Control ≥ 2 or ≤ 0.5, *p* ≤ 0.01) in the hair between Alzheimer’s disease and controls. (**A**) 67 of 12,353 detected signals were discriminatory features (fold change ≥ 2 or ≤ 0.5, *p* ≤ 0.01) in positive ion modes. Of these, abundances of 33 signals were increased and 38 signals were decreased in AD group relative to control in positive ion mode. (**B**) 5 of 8821 signals were significant, using the criteria (fold change ≥ 2 or ≤ 0.5, *p* ≤ 0.01) in negative ion mode. Of those, 2 signals were overexpressed, and 3 signals were under expressed in AD patients relative to control in negative ion mode analysis. The horizontal and the vertical red dashed lines represent the cutoff of −Log10(*p*-value) and Log2 (AD / Control) for 2 and ≧ 1 or ≦−1, respectively.

As shown in Figure 2A,B, two volcano plots were generated to reveal significant signal intensity changes (|log2 (fold-change)|  ≥ 1 and *p* value ≤  0.01) in peak features between the AD and control groups in positive and negative UHPLC-HRMS ion modes, revealing 72 discriminatory features (Table S1). Among these, 32 peak features indicated overexpression and 40 indicated low expression in AD patients relative to the levels in the control subjects. The RSDs of the peak areas of the 72 discriminatory peak features in the QC sample and 12 replicates are shown in Figure S1B, indicating that 61 discriminatory peak features had RSD values below 50%. Subsequently, these discriminatory features were subjected to tandem mass spectrometry (MS/MS) analysis using the parallel reaction monitoring (PRM) method for chemical structure identification.

Sixty-one discriminatory features were discovered by our untargeted metabolomic strategy, and an additional analytical run using the PRM method for MS/MS fragmentation under 50% normalized collision energy was performed in both positive and negative ion modes. The chemical identification of the metabolites in the discriminatory features was achieved using MS-DIAL 4.48 and MS-FINDER 3.50. We used MS-DIAL 4.48 software to perform mass spectral similarity searches of experimental MS/MS results in the Massbank of North America (MoNA) database. The compound with the highest total score value, calculated based on MS1 similarity, isotopic similarity and MS/MS similarity above 70%, was assigned to the feature. The chemical structures of ten biomarkers were identified through the MoNA database with MS-DIAL 4.48. In addition, in order to comprehensively identify the remaining chemical structures of the discriminatory features, we matched the experimental MS/MS spectra against a variety of MS-FINDER 3.50-generated fragments calculated from candidate compounds retrieved from chemical compound databases, such as PubChem and Human Metabolome Database. The tentative chemical compound with the highest structure score value above 5.0 (out of 10.0) was assigned to the discriminatory feature. In addition, the unequivocal molecular formulas were annotated utilizing MS-FINDER 3.50. However, in some cases, the experimental MS/MS spectrum did not match with either the MoNA or MS-FINDER-generated fragments. The cutoff for formula score was set at 3.0 (out of 5.0). A total of 21 biomarkers were identified through the MS/MS spectra generated by MS-FINDER 3.50 (Table S2).

**Figure 3:**
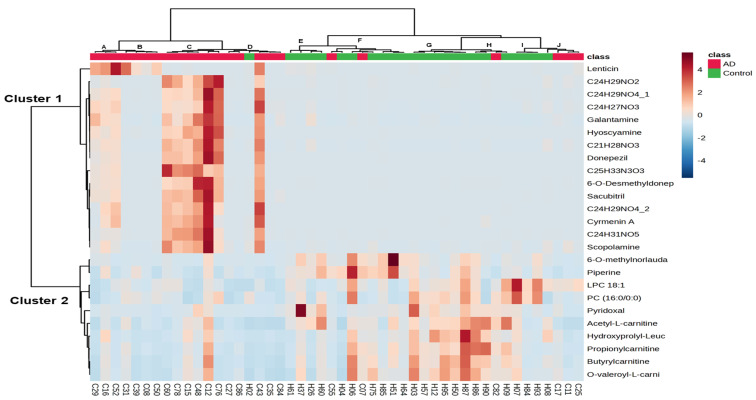
Two-way hierarchical clustering analysis (HCA) of 25 annotated metabolites in 48 hair samples. The rows represent 25 discriminatory metabolites whose p values are lower than 0.01 between patients with AD and controls, and the columns represent 48 hair samples. The z-score value of each feature among 48 hair samples is plotted in a red–blue color scale. The red color indicates that the abundances of the feature are higher than its average abundance among 48 samples, and bright blue indicates its abundances are approximately equal to its average abundance among 48 samples. Conversely, blue indicates its abundances are lower than its average abundance among 48 samples. The 48 hair samples could be grouped into 10 clusters (Clusters A~J) with similar peak feature profiles, and 25 annotated metabolites could be primarily grouped into 2 clusters (Clusters 1 and 2).

To find the discriminatory features with similar expression patterns in the 48 hair samples, unsupervised clustering of the detected normalized intensity of 25 annotated metabolites was performed by two-way hierarchical cluster analysis (HCA) based on Pearson’s correlation. Figure 3 shows a heatmap with z score values, which indicate the number of standard deviations above or below the average value for each raw value, where the relative abundance of metabolites detected in each sample is represented using a color scale. Each row represents a discriminatory metabolite, and each column represents a biological sample. The z score value of each feature is plotted on a red‒blue color scale. A red color indicates that the z score value is greater than 0, and a blue color indicates that the z score is lower than 0. The 25 annotated metabolites were grouped into 2 clusters (Clusters 1 and 2). Cluster 1 metabolites showed increased levels in AD patients compared to controls, and cluster 2 metabolites, including acetyl-L-carnitine, propionylcarnitine, butyrylcarnitine, O-valeroyl-L-carnitine, PC (16:0/0:0), LPC 18:1, piperine, pyridoxal, 6-O-methylnorlaudanosoline, and hydroxyprolyl-leucine, showed decreased levels in patients with AD compared to those in healthy controls. The 48 hair samples could be grouped into 10 clusters (Clusters A~J) based on 25 discriminatory metabolites with similar peak feature profiles. Clusters E to J primarily consisted of the cognitively healthy controls. Clusters C and D primarily included the 7 AD patients with a CDR score of 0.5 and the 4 AD patients with a CDR score of 1, suggesting that the levels of these metabolites might be used to group patients with questionable AD.

### 2.4. Application of Biomarkers for AD Diagnosis

**Figure 4:**
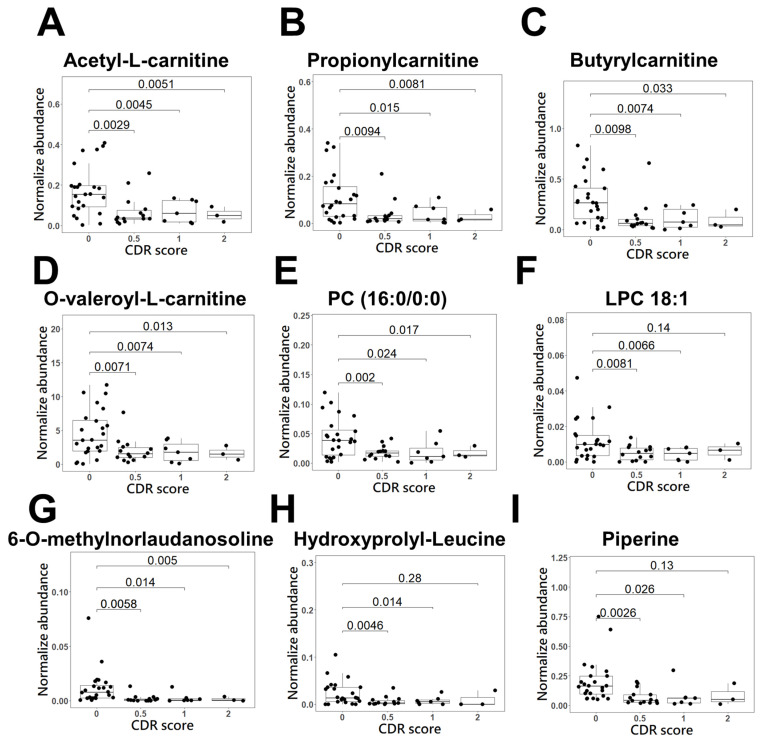
Scatter and box plots of candidate biomarker metabolites for early detection of AD dementia in the derived panel. The metabolites, (**A**) acetyl-L-carnitine, (**B**) propionylcarnitine, (**C**) butyrylcarnitine, (**D**) O-valeroyl-L-carnitine, (**E**) PC (16:0/0:0), (**F**) LPC 18:1, (**G**) 6-O-methylnorlaudanosoline, (**H**) hydroxyprolyl-leucine, and (**I**) piperine, represent the normalized metabolites intensity.

**Figure 5:**
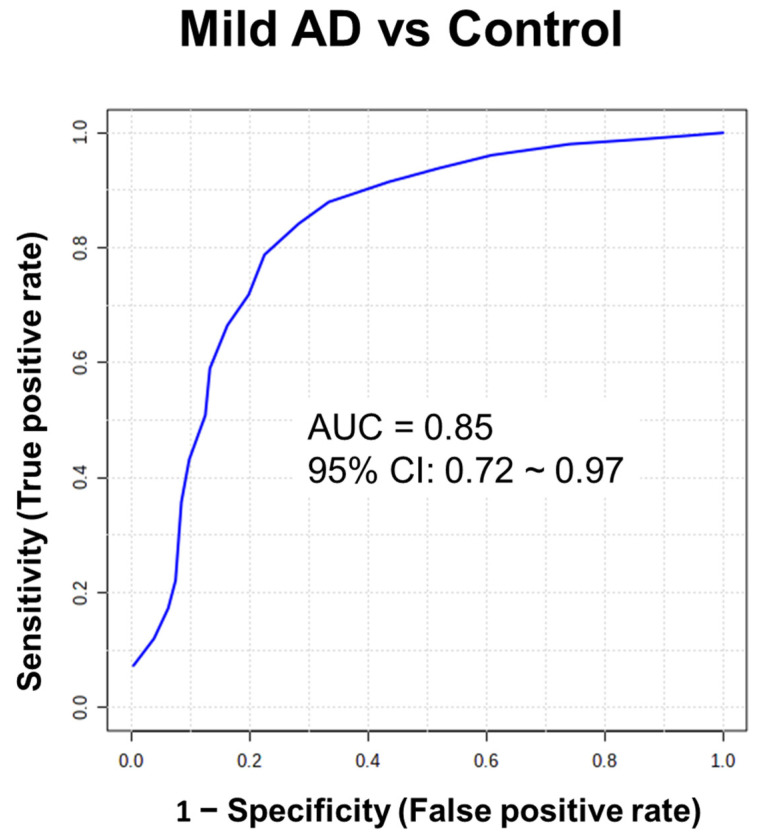
Receiver operator characteristic (ROC) curve analysis of statistically significant metabolites in the hair between patients with AD (CDR score of 0.5) and controls. ROC curve analysis was performed to compare the predictive power of early stage of AD and control with a combination of 9 metabolites using random forest.

We further investigated the normalized peak area of the 25 discriminatory metabolites in patients with different severities of AD and the healthy control group using Student’s *t* test (Figure 4 (A~J)). The different severities of AD are evaluated by CDR, which assesses cognitive, behavioral, and functional aspects of AD [24]. CDR scores of 0.5, 1, 2, and 3 indicate mild cognitive impairment (MCI), mild AD, moderate AD, and severe AD, respectively [25]. It was revealed that 9 metabolites had significant differential levels between AD patients with a CDR score of 0.5 and healthy controls (*p* < 0.01), indicating high potential for identifying the initiation or promotion phase of AD dementia in the early stage. The receiver operating characteristic (ROC) curves of these 9 biomarker candidates between 14 patients with AD (CDR score of 0.5) and controls were plotted to further evaluate their performance and calculate the area under the curve (AUC). These results are shown in Table 2, and the AUC values of the 9 metabolite biomarkers ranged from 0.73 to 0.86. Among these compounds, 6-O-methylnorlaudanosoline reached 90% sensitivity and 80% specificity in mild AD. Acetyl-L-carnitine and butyrylcarnitine were found to indicate AD in patients with approximately 80% sensitivity and at least 80% specificity. Propionylcarnitine and O-valeroyl-L-carnitine had approximately 70% sensitivity and specificity. Using a composite panel of these 9 biomarker candidates, the AUC value was 0.85 (95% CI: 0.72~0.97) in patients with AD with a CDR score of 0.5 compared to healthy controls. The combined diagnostic sensitivity and specificity of the composite panel in AD patients were 86% and 71%, respectively (Figure 5), suggesting that these 9 biomarker candidates may be used for the early detection of AD.

Biomarkers that are objective and quantifiable characteristics of biological processes or conditions have emerged as important components in the search for the pathogenesis of AD and might contribute to pharmacotherapeutic breakthroughs. In addition, due to the apparent evidence that alterations in the levels of biomarkers might precede disease phenotypes by decades, further elucidation of these medical signs may also pave the way for prevention. We then further investigated the associations between these 9 potential biomarkers and MoCA scores, which are general mental status scales used to evaluate cognitive functions. Figure S2 shows that the correlation coefficient values were 0.45 (*p* = 0.0014), 0.44 (*p* = 0.0021), 0.44 (*p* = 0.0016), 0.43 (*p* = 0.0019), and 0.40 (*p* = 0.0064) between MoCA scores and acetyl-L-carnitine, propionyl carnitine, butyrylcarnitine, O-valeroyl-L-carnitine, and 6-O-methylnorlaudanosoline, respectively.

## 3. Discussion

Chemical substances secreted from tissues or organs into the bloodstream in response to different physiological needs or stresses are incorporated into the hair matrix during its growth [20,26,27,28,29]. Hair is an analytical biospecimen for the long-term accumulation of both xenobiotic exposure and endogenous perturbations, which may reflect the integral metabolic profiles of body burden over several months. Hair is not a homogeneous matrix but consists of keratinized cells that form three concentric structures: cuticle, cortex, and medulla. As the cells of hair follicles die and fuse to form hair strands, the chemical compounds are retained and accumulate in this extremely stable structure. Twenty-five discriminatory metabolites were discovered in hair between AD patients and controls using an HRMS-based untargeted metabolomics approach with good reproducibility, suggesting that the hair matrix might emerge as a potential biospecimen for biomarker discovery. Moreover, if the levels of these biomarkers could be determined by a multiple reaction monitoring (MRM) method using targeted metabolomics to provide the cutoff for biomarker levels, biomarkers in human hair could provide a sensitive and noninvasive approach for AD diagnosis. Donepezil and galantamine, which are treatments for AD dementia, showed increased levels in the hair of AD patients compared to controls. These drugs enable central cholinergic activity by inhibiting the physiological breakdown of acetylcholine by the enzyme acetylcholinesterase in synaptic gaps [30]. In addition to the detection of donepezil, increased levels of 6-desmethyldonepezil, a metabolite of donepezil, were detected in the hair of AD patients compared to the controls in this study.

We discovered that the combination of five metabolites, 6-O-methylnorlaudanosoline, acetyl-L-carnitine, pro-pionylcarnitine, butyrylcarnitine, and O-valeroyl-L-carnitine, in hair could contribute to distinguishing mild AD patients from controls (Figure 5), suggesting that they might be early markers of AD. A panel of biomarkers is likely to compensate for the deficient sensitivity and specificity of a single marker used to distinguish AD patients from controls [9,31,32]. For example, when glutamine is combined with valine, a higher AUC value is generated, leading to an increased predictive power superior to that of glutamine or valine alone [31]. In our data, the discriminating performance of combining four acylcarnitines and 6-O-methylnorlaudanosoline was superior to that of any acylcarnitine alone. The association between acyl-carnitine concentrations in hair and AD pathology are not yet fully understood, although alterations in their concentrations in plasma and CSF have previously been reported. Decreased levels of acylcarnitines in plasma and CSF have been observed in AD patients [9,33,34]. Acyl-carnitine can disrupt fatty acid transport into mitochondria for β-oxidation and further perturbations of energy metabolism in the brain, which is consistent with our observation that acyl-carnitines showed a lower expression level in AD patients than in healthy subjects. An association between AD and dysregulation of lipid metabolism, particularly perturbations in brain membrane lipids, has been implicated in AD pathology. The increased level of glycerophosphocholine and choline generated by hydrolysis of PC in the CSF of patients with AD compared to healthy controls has been reported [35,36]. Previous studies have shown that the levels of PC (16:0/0:0) and LPC 18:1 are significantly lower in patients with AD than in healthy subjects [37,38]. Moreover, it is suggested that alterations in the metabolism of choline-containing phospholipids in the brain are closely associated with membrane changes in AD, which is consistent with our findings that reductions in PC (16:0/0:0) and LPC 18:1 levels were observed in AD patients compared to controls [39]. Studies showed lower levels of piperine in the blood of patients with AD than in that of healthy controls [40]. Piperine, the primary alkaloid constituent of black pepper, has been reported to attenuate cognitive impairment in an experimental mouse model of sporadic Alzheimer’s disease [41].

In addition to our observations of evident differences between AD and controls concerning acylcarnitines, phosphatidylcholines, and piperine, we also observed the novel AD biomarker 6-O-methylnorlaudanosoline. The proposed mechanism by which 6-O-methylnorlaudanosoline influences AD pathogenesis involves a reduction in oxidative stress in the brain. It has been reported that 6-O-methylnorlaudanosoline, a derivative of norlaudanosoline, reacts through catechol-O-methyltransferase in the brain [42]. The methylation of norlaudanosoline can increase its hydrophobicity and allow it to enter the hair matrix through blood via passive diffusion. Norlaudanosoline, a dopaminergic neurotoxin, increases intracellular levels of reactive oxygen species (ROS) [43,44], contributing to oxidative stress-induced synaptic dysfunction in AD pathogenesis. In the brains of AD patients, oxidative stress signatures are observed at the very early stage of the disease [45,46]. The neurotoxicity of norlaudanosoline may result from its tendency to autoxidize and yield reactive quinoids and subsequent hydroxyl radicals by redox cycling, further leading to oxidative stress-related cell death and DNA damage [47]. Moreover, norlaudanosoline, derived from dopamine through condensation with 3,4-dihydroxyphenylacetaldehyde (dopaldehyde), is considered a dopaminergic neurotoxin and has been implicated in the pathology of Parkinson’s disease [48,49]. It has been reported that norlaudanosoline, detected in the urine of patients with Parkinson’s disease treated with L-3,4-dihydroxyphenylalanine (L-DOPA), reduces tyrosine hydroxylase activity during L-DOPA production [50,51]. Mono-O-methylated norlaudanosoline reduced the in vitro production of hydroxyl radicals during autoxidation in previous studies [52,53]. Enzyme-mediated methylation reactions in the brain play vital roles in cells exposed to neuroprotective and neurotoxic components. The reduction in 6-O-methylnorlaudanosoline levels in AD patients might indicate that norlaudanosoline primarily undergoes spontaneous reactions to yield reactive quinoids and hydroxyl radicals, enhancing oxidative stress-induced neuronal apoptosis and dopamine degeneration. Therefore, 6-O-methylnorlaudanosoline may contribute to the underlying AD pathogenesis and could be used for early detection of AD in hair.

Some limitations of our study should be considered. In some experiments, the severity of phenotypes was not measured, such as the measurement of tau neurofibrillary or Aβ plaques. AD patients were diagnosed only through cognitive examination by professional neurological physicians. That is, AD was diagnosed based on observational evaluation of behavioral symptoms and memory impairment, but early symptoms of AD are similar to those of other neurological disorders. In general, abnormal Aβ and tau levels are used to identify AD; however, the measurement of Aβ and tau in the CSF or through the use of positron-emission tomography imaging involves a complex sampling procedure and is expensive. Certain discriminatory features could not be identified as known metabolites or chemical formulas, creating a significant bottleneck in untargeted metabolomics research. Using the mass spectral database MoNA and the spectrum prediction program MS-FINDER 3.50, we identified the chemical structure of 40% of discriminatory features; however, more than 60% of the unknown chemical compounds might be important biomarkers of diseases. The spectrum of all chemicals was not recruited in the spectrum database due to the lack of authentic chemical standards available and endogenous metabolites. This makes it extremely difficult to annotate and interpret HRMS-based metabolome data. We recognize that not all polar metabolites were comprehensively identified; however, there were two important reasons that only reverse-phase liquid chromatography (RPLC) was employed. Perturbations in amino acids, bioenergetic metabolism, and redox imbalance are commonly observed in AD [3,9,54]. Hydrophilic metabolites in the relevant pathways were generally detected using a hydrophilic interaction liquid chromatography (HILIC) column. If an analytical method for detecting hydrophilic metabolites in human hair can be developed, a combination of RPLC and HILIC can ensure the global coverage of metabolites in an untargeted metabolomics investigation designed for AD biomarker discovery. Nine biomarkers for AD were discovered in human hair in this study, although the practical diagnostic accuracy of these biomarkers in hair requires further investigation and validation. In addition, the chemical structures of the biomarkers were only identified through a spectral database using MS-DIAL 4.48 or annotated by MS-FINDER 3.50. If more patients with AD and controls are recruited for biomarker validation, this might become an excellent method for detecting early AD in a noninvasive manner. Alterations in the metabolites across AD development could not be demonstrated in this study since a cross-sectional design was used. However, a longitudinal study design that involves repeated observations of the same participants for an extended period can be performed. In that case, the biomarkers for AD development might contribute to recognizing the causes of AD and developing a novel therapeutic approach.

## 4. Materials and Methods

### 4.1. Participant Description

The 48 participants included adults 40 years or older without a history of stroke or known malignancy. AD patients (n = 24) were recruited from the Department of Neurology in National Cheng Kung University Hospital, Tainan, Taiwan, and were diagnosed by professional physicians. The Montreal Cognitive Assessment (MoCA) was performed, and the severity of the dementia symptoms was assessed by neurologists using the Clinical Dementia Rating (CDR). Age- and sex-matched control subjects (n = 24) were recruited from the Department of Family Medicine in National Cheng Kung University Hospital, Tainan, Taiwan, and underwent MoCA, with a MoCA score ≥ 26 points. Other important information on age, body mass index (BMI), cosmetic hair treatment, smoking status, and alcohol consumption was collected and is shown in Table 1. This study was approved by the Ethics Committee of National Cheng Kung University Hospital. All participants in the study signed a written informed consent form under the rules and requirements of the Institutional Review Board of National Cheng Kung University Hospital (IRB approval no. B-ER-108-188).

### 4.2. Hair Sampling and Metabolite Extraction

Hair was cut 0.5 cm away from the scalp with scissors, secured in aluminum foil and stored at 4 °C until further analysis. The washing procedure was performed according to the Society of Hair Testing guidelines as a mandatory step to remove contaminants deposited on the hair shaft [55]. A six-milligram 3-cm hair sample was weighed in a glass tube and washed with 1.8 mL acetone followed by 1.8 mL deionized water in an ultrasonic bath for 2 min each. The washing solutions were then discarded, and the samples were dried under nitrogen for 90 min. Afterward, the dried hair samples were cut into round 0.2-cm snippets with a pair of scissors and subjected to the extraction procedure developed in the previous study [22]. The samples were mixed with 300 μL of methanol (MeOH)/phosphate-buffered saline (PBS) (50/50, *v*/*v*) and sonicated for 4 h at 55 °C. The extracts were centrifuged at 15,000 ×g for 15 min, and the supernatants were collected. The hair extract was subsequently evaporated to dryness by a speed vac, and the residue was reconstituted with 30 μL of MeOH/H_2_O (50/50, *v*/*v*). A quality control (QC) sample was prepared by pooling 5 mg of the hair samples from each of the 48 participants and subjected to the extraction procedure. QC samples were prepared in parallel with study samples and analyzed after 4 sample injections to monitor the robustness of the large-scale analysis.

### 4.3. UHPLC-HRMS Analysis

A UHPLC system coupled with an Orbitrap HRMS system (Thermo Fisher Scientific, Bremen, Germany) was used for hair sample analysis. Chromatographic separation was performed by a Waters Acquity UPLC BEH C18 column (2.1 mm × 100 mm, 1.7 μm, Waters, Milford, MA, USA). The mobile phases were composed of (A) 2% ACN in deionized water with 0.1% formic acid and (B) acetonitrile with 0.1% formic acid. The gradient conditions were as follows: 0–1 min, 2% B; 1–11 min, 2–99% B; 11–13 min, 99% B; 13–13.01 min, 99–2% B; 13.01–14 min, 2% B. The flow rate was set at 250 μL/min. The column temperature was kept at 40 °C, and the injection volume was 5 μL.

The Q Exactive Orbitrap and Lumos Orbitrap were operated in positive and negative modes, respectively. The mass range was *m*/*z* 100 to 1000, and the resolution in the full scan method was 70,000 and 60,000 using the Q Exactive Orbitrap and Lumos Orbitrap, respectively.

### 4.4. Data Processing, Statistical Analysis, and Metabolite Identification

The raw data were analyzed by Progenesis QI software (Waters, Milford, MA, USA), performing peak detection and alignment. Peak tables containing accurate masses, retention times, and peak abundances were exported. The features with S/N < 3 were considered as the absence of peaks and were filtered out. Before performing the univariate analysis to discover the differential features, each raw abundance was normalized by dividing by the sum of the raw abundances of all peaks in the corresponding sample. Volcano plots were generated by R 4.2.2 to distinguish the discriminatory features. We generated a heatmap with unsupervised HCA of the 25 annotated discriminatory metabolites based on z scores using Pearson correlation by MetaboAnalyst 5.0 to visualize the clustering pattern of the 25 metabolites in the hair samples [56]. Discriminatory features were selected for tandem mass spectrometry (MS/MS) analysis by the PRM method at a resolution of 17,500 by high energy collisional dissociation (HCD) with a normalization collision energy of 50% using a Thermo Fusion Lumos Orbitrap mass spectrometer (Thermo Fisher Scientific, San Diego, CA, USA), operated in both positive and negative ion modes with the same chromatographic conditions described in the previous section. We performed mass spectral similarity searches in the experimental MoNA database (https://mona.fiehnlab.ucdavis.edu/, assessed on 10 September 2021), which was downloaded on 10 September 2021 in MS-DIAL 4.48, and in silico generated fragments calculated from candidates retrieved from available compound databases in MS-FINDER 3.50 [57,58]. Using MS-DIAL 4.48 for chemical identification, the search windows were set with a mass tolerance of 5 ppm for the precursor masses, and the cutoff value of the total score was set at 70%. Using MS-FINDER 3.50 for chemical identification, the search windows were set with a mass tolerance of 5 ppm for the precursor masses, and the cutoff for formula and structure score were set at 3.0 (out of 5) and 6.0 (out of 10), respectively, according to our previous research [22]. Receiver operating characteristic (ROC) curves were plotted using MetaboAnalyst 5.0 to evaluate the predictive performance of the model, and random forest analysis was performed to generate a metabolite panel [56].

## 5. Conclusions

The nine discriminatory metabolites highlighted here may be used as biomarkers for early detection of AD dementia or for therapeutic development after validation in a larger cohort. Our study provides evidence that the hair metabolome shows a similar trend with the pathology of AD and has great potential to reflect the long-term perturbations of oxidative stress, dopaminergic neurotoxins, mitochondrial β-oxidation metabolism, and dysregulation of lipids in AD. Investigation into the specific roles of the identified metabolic changes in the pathogenesis of AD will offer novel insight into metabolic alterations. In addition, if validated in an external cohort of patients, the identified biomarker panel offers a promising method to detect the early stage of Alzheimer’s disease in a noninvasive manner.

## Figures and Tables

**Table 1:** Demographics of Alzheimer’s disease (AD) patients and control subjects.

Characteristic	AD Patients	Healthy Control	*p*-Value
Sex			
Female	18	18	
Male	6	6	
Age (years)			
Mean ± SD	68.7 ± 6.5	66.2 ± 6.2	0.17
BMI (kg/m^2^)			
Mean ± SD	24.2 ± 4.0	23.9 ± 3.2	0.83
MoCA score			
Mean ± SD	15.5 ± 5.3	27.4 ± 1.4	<0.0001
CDR score			
0.5	14	-	-
1	7	-	
2	3	-	
Cosmetic hair treatment			
Never	13	15	0.93
Perming	3	3	
Dyeing	8	6	
Both	2	2	
Smoking status			
Never	21	20	0.33
Past	3	2	
Current	0	2	
Alcohol consumption			
Never	23	22	0.60
Past	0	1	
Current	1	1	

Note: Student’s *t* test was applied to calculate the statistical significance of age, BMI and MoCA score between AD and controls, and Chi-square test was used to calculate the statistical significance of cosmetic hair treatment, smoking status and alcohol consumption.

**Table 2:** Statistically significant metabolites between 14 AD patients (CDR score of 0.5) and 24 controls using untargeted metabolomics analysis.

PubChem CID	Metabolite Name	Mild AD/Control ^(a)^	*p* Value ^(b)^	AUC ^(c)^
182440	6-O-methylnorlaudanosoline	0.16	0.0056	0.86
638024	Piperine	0.36	0.0026	0.82
213144	Butyrylcarnitine	0.40	0.0098	0.78
7045767	Acetyl-L-carnitine	0.41	0.0029	0.77
107738	Propionylcarnitine	0.36	0.0094	0.75
21777566	Hydroxyprolyl-Leucine	0.27	0.0046	0.75
24779465	LPC 18:1	0.41	0.0081	0.74
460602	PC (16:0/0:0)	0.42	0.0020	0.73
16226475	O-valeroyl-L-carnitine	0.45	0.0071	0.73
1050	Pyridoxal	0.35	0.0230	0.73

Note: ^(a)^ Average of peak abundance of metabolites in AD patients (CDR score of 0.5) divided by that in controls ^(b)^ All measurements were analyzed by Student *t*-test and *p*-value < 0.01 indicates a statistically significant difference ^(c)^ AUC of AD patients (CDR score of 0.5) versus controls.

## Data Availability

The data presented in this study are available in the article and Supplementary Material.

## Supplementary Materials

The following supporting information can be downloaded at: https://www.mdpi.com/article/10.3390/molecules28052166/s1, Figure S1. Quality control (QC) of the analytical method. (A) RSD distribution of all the detected peak features in the QC samples. Red line represents the 85% of all detected peaks. (B) RSD distribution in the QC sample of the discriminatory peak features between AD and controls. Red line represents 90% of the discriminatory peak features. Figure S2: Correlations between 10 metabolites and MoCA score. Table S1. The 72 discriminatory features analyzed by fold change and Student’s *t* test. Table S2. Chemical structures were annotated using MoNA and MS-FINDER 3.50.

## Sample Availability

Samples of the compounds are available from the authors.
