# Supplementary material for: Discovering Hair Biomarkers of Alzheimer’s Disease Using High Resolution Mass Spectrometry-Based Untargeted Metabolomics
Source: Molecules. 2023 Feb 25;28(5):2166. doi: 10.3390/molecules28052166 (PMC10004788; doi:10.3390/molecules28052166)
Supplement: Supplementary file 1 [file molecules-28-02166-s001.zip › molecules-2198881-supplementary.pdf]

Supplementary material

Discovering hair biomarkers of Alzheimer's disease using high resolution mass spectrometry-based untargeted metabolomics

Yu-Hsiang Su [1], Chih-Wei Chang [2, †], Jen-Yi Hsu [2], Shih-Wen Li [2], Pi-Shan Sung [3], Ru-Hsueh Wang [4], Chih-Hsing Wu [4,5], Pao-Chi Liao [2,6, *]

1. Division of Neurology, Department of Internal Medicine, Ditmanson Medical Foundation Chia-Yi Christian Hospital, Chiayi City 60002, Taiwan
2. Department of Environmental and Occupational Health, College of Medicine, National Cheng Kung University, Tainan, 704, Taiwan
3. Department of Neurology, National Cheng Kung University Hospital, College of Medicine, National Cheng Kung University, Tainan, 704, Taiwan
4. Department of Family Medicine, National Cheng Kung University Hospital, College of Medicine, National Cheng Kung University, Tainan, 704, Taiwan
5. Institute of Gerontology, College of Medicine, National Cheng Kung University, Tainan,701, Taiwan
6. Department of Food Safety/Hygiene and Risk Management, College of Medicine, National Cheng Kung University, Tainan, 701, Taiwan

† The author contributed equally to this work.

*Corresponding author:

Dr. Pao-Chi Liao

Department of Environmental and Occupational Health, College of Medicine,

National Cheng Kung University, 138 Sheng-Li Road, Tainan, 704, Taiwan,

Tel: 886-6-2353535 ext 5566

Fax: 886-6-2752484

Email: liaopc@mail.ncku.edu.tw

Figure content

Figure S1. Quality control (QC) of the analytical method. (A) RSD distribution of all the detected peak features in the QC samples. Red line represents the 85% of all detected peaks. (B) RSD distribution in the QC sample of the discriminatory peak features between AD and controls. Red line represents 90% of the discriminatory peak features.

Figure S2. Correlations between 10 metabolites and MoCA score (A) acetyl-L-carnitine, (B) propionylcarnitine, (C) butyrylcarnitine, (D) O-valeroyl-L-carnitine, (E) 6-O-methylnorlaudanosoline, (F) PC (16:0/0:0), (G) LPC 18:1, (H) pyridoxal, (I) hydroxyprolyl-leucine, and (J) piperine**.**

Table content

Table S1. The 72 discriminatory peak features analyzed by fold change and Student's t test.

Table S2. Chemical structures annotated by MS-FINDER and MoNA within MS-DIAL.

Table S1. The 72 discriminatory peak features analyzed by fold change and Student's t test.

Peak No.	Retention time (min)	m/z	AD / Control	P
#01	0.81	134.0762	0.49	0.0091
#02	7.89	145.0647	12.46	0.0095
#03	0.84	149.5973	0.38	0.0050
#04	1.74	150.0549	0.28	0.0077
#05	1.25	198.037	0.47	0.0039
#06	4.68	199.0865	0.48	0.0010
#07	0.98	204.1228	0.40	0.0005
#08	4.43	213.1231	0.33	0.0084
#09	1.90	218.1384	0.35	0.0035
#10	1.08	218.1385	0.30	0.0028
#11	2.52	229.1178	0.31	0.0036
#12	4.47	232.1541	0.38	0.0019
#13	4.43	235.1072	0.30	0.0089
#14	9.63	240.1955	0.24	0.0058
#15	1.04	245.1493	0.30	0.0041
#16	5.12	246.1593	0.42	0.0077
#17	5.11	246.1696	0.43	0.0019
#18	1.57	247.1284	0.46	0.0093
#19	7.89	247.1437	16.98	0.0100
#20	5.11	248.1749	0.41	0.0020
#21	8.71	261.1458	0.34	0.0031
#22	4.07	267.0953	0.40	0.0080
#23	6.39	268.1151	6.75	0.0097
#24	8.44	272.1276	0.26	0.0010
#25	1.26	272.1963	0.44	0.0069
#26	4.50	274.1756	0.41	0.0094
#27	5.07	276.1589	7.19	0.0074
#28	2.32	281.1126	4.75	0.0051
#29	8.93	286.1431	0.35	0.0020
#30	9.06	286.1431	0.37	0.0020
#31	9.14	286.1431	0.34	0.0018
#32	5.71	288.1590	10.12	0.0021
#33	5.56	290.1745	179.00	0.0030
#34	5.56	292.1807	10.67	0.0075
#35	7.15	302.1379	0.17	0.0042

#36	7.28	302.1379	0.21	0.0044
#37	7.28	303.1415	0.10	0.0059
#38	6.24	304.1538	6.47	0.0087
#39	9.77	312.1586	0.26	0.0093
#40	6.25	327.1368	6.48	0.0099
#41	10.90	329.2467	0.39	0.0082
#42	4.98	344.1233	4.86	0.0070
#43	6.68	353.2216	0.39	0.0000
#44	7.34	364.2262	18.06	0.0097
#45	6.69	365.1974	11.61	0.0046
#46	6.21	366.2056	77.20	0.0065
#47	11.21	366.2994	0.39	0.0010
#48	9.04	367.2374	0.45	0.0011
#49	6.61	378.2056	17.77	0.0054
#50	6.69	379.9793	87.88	0.0047
#51	6.42	380.2211	24.93	0.0074
#52	6.69	380.2211	58.60	0.0030
#53	5.69	382.2005	18.49	0.0065
#54	5.95	382.2005	17.46	0.0084
#55	6.69	382.2274	69.98	0.0032
#56	6.51	382.2368	89.53	0.0040
#57	9.14	385.2473	0.35	0.0018
#58	8.94	385.2474	0.32	0.0005
#59	6.13	396.2161	135.20	0.0051
#60	6.51	396.2162	123.54	0.0064
#61	6.13	398.2222	17.78	0.0082
#62	5.56	407.2531	43.01	0.0056
#63	6.01	412.2109	68.92	0.0029
#64	6.45	414.2266	94.70	0.0069
#65	6.18	424.2583	252.94	0.0024
#66	8.80	481.3044	0.34	0.0081
#67	10.66	496.3388	0.42	0.0013
#68	6.70	497.2996	10.55	0.0028
#69	10.66	497.3419	0.38	0.0056
#70	6.13	513.2948	664.05	0.0044
#71	10.92	522.3543	0.40	0.0046
#72	6.71	795.4117	270.16	0.0032

Table S2. Chemical structures annotated by MS-FINDER and MoNA within MS-DIAL.

Compound name	Formula	m/z	Retention time (min)	ID method	MS-DIAL match score	MS-FINDER Formula score	MS-FINDER structure score
L-Acetylcarnitine	C9H17NO4	204.1228	0.98	MoNA/MS-Finder	71.8	3.66	6.21
Hydroxyprolyl-Leucine	C11H20N2O4	245.1493	1.04	MS-Finder		3.60	
Pyridoxal	C8H9NO3	150.0549	1.74	MoNA	79.3		
Propionylcarnitine	C8H17NO4	218.1384	1.90	MS-Finder		3.93	6.7
Butyryl-carnitine	C11H21NO4	232.1541	4.47	MoNA/MS-Finder	95.6	3.55	5.91
Valeryl-carnitine	C12H23NO4	246.1696	5.11	MoNA/MS-Finder	90.8	3.55	5.99
Hyoscyamine	C17H23NO3	290.1745	5.56	MoNA/MS-Finder	81.8	3.61	6.98
Cyrmenin A	C22H34N2O5	407.2531	5.56	MS-Finder		3.42	
Galantamine	C17H21NO3	288.1590	5.71	MS-Finder		3.54	
Sacubitril	C24H29NO5	412.2109	6.01	MS-Finder		3.85	
	C24H29NO4	396.2161	6.13	MS-Finder		3.70	
	C25H33N3O3	424.2583	6.18	MS-Finder		3.02	
6-O-Desmethyldonepezil	C23H27NO3	366.2056	6.21	MS-Finder		4.05	7.18
Scopolamine	C17H21NO4	304.1538	6.24	MS-Finder		3.74	7.19
	C24H31NO5	414.2266	6.45	MS-Finder		3.77	
2-benzoyl-1-(2,5-dimethoxyphenyl)-decahydroisoquinolin-4a-ol	C24H29NO4	396.2162	6.51	MS-Finder		3.69	
	C24H27NO3	378.2056	6.61	MS-Finder		3.58	
	C21H28NO3	365.1974	6.69	MS-Finder		3.23	

Donepezil	C24H29NO3	380.2211	6.69	MoNA/MS-Finder	92.1	3.83	7.25
6-O-methylnorlaudanosoline	C17H19NO4	302.1379	7.15	MS-Finder		3.78	
	C24H29NO2	364.2262	7.34	MS-Finder		3.26	
Lenticin	C14H18N2O2	247.1437	7.89	MoNA/MS-Finder	95.8	3.36	5.71
Piperine	C17H19NO3	286.1431	9.14	MoNA/MS-Finder	94.8	3.69	6.59
PC(16:0/0:0)	C24H50NO7P	496.3388	10.66	MoNA	88.6		
LPC 18:1	C26H52NO7P	522.3543	10.92	MoNA	75.2		

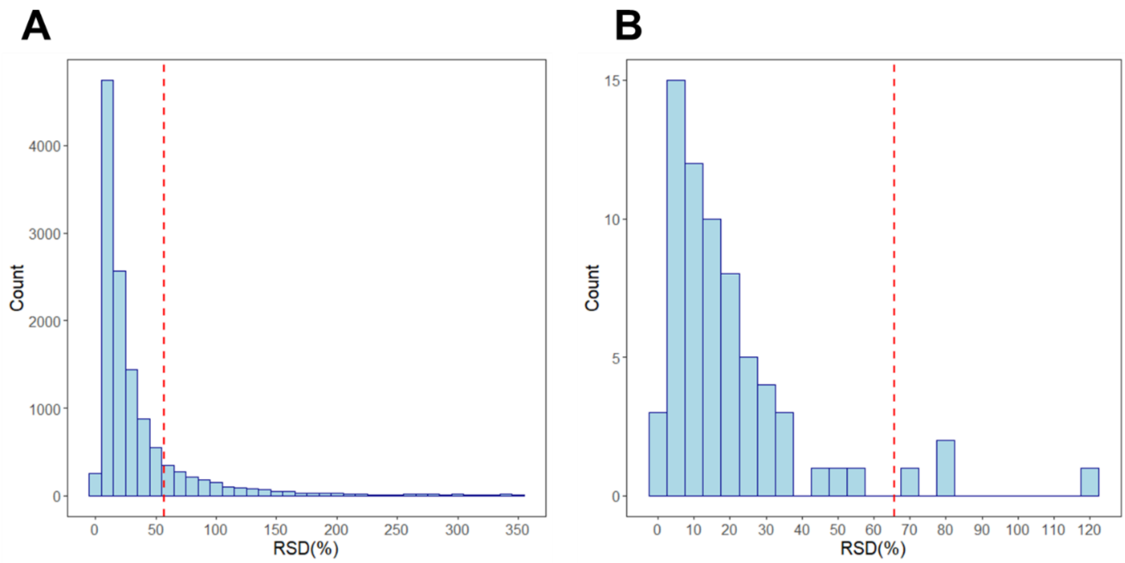

Figure S1. Quality control (QC) of the analytical method. (A) RSD distribution of all the detected peak features in the QC samples. Red line represents the 85% of all detected peaks. (B) RSD distribution in the QC sample of the discriminatory peak features between AD and controls. Red line represents 90% of the discriminatory peak features.

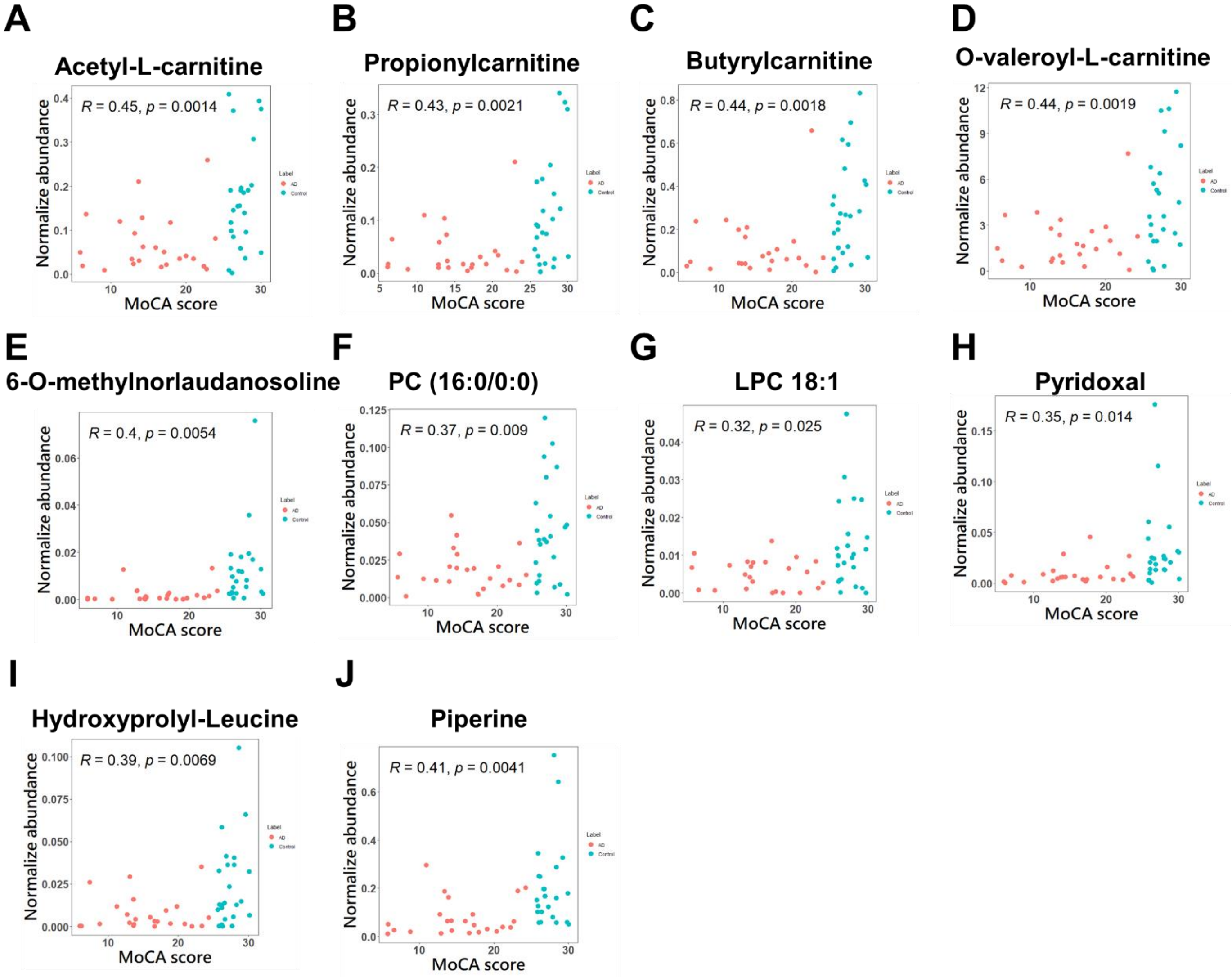

Figure S2. Correlations between 10 metabolites, (A) acetyl-L-carnitine, **(B)** propionylcarnitine, **(C)** butyrylcarnitine, **(D)** O-valeroyl-L-carnitine, **(E)** 6-O-methylnorlaudanosoline, **(F)** PC (16:0/0:0), **(G)** LPC 18:1, **(H)** pyridoxal, **(I)** hydroxyprolyl-leucine, and **(J)** piperine, and MoCA score.
